# Association of the *ADRB2* rs1042714 variant with retinopathy of prematurity highlights the importance of the renin-angiotensin-aldosterone system

**DOI:** 10.1038/s41598-025-95055-1

**Published:** 2025-04-02

**Authors:** Anna Chmielarz-Czarnocińska, Anna Durska, Bartosz Skulimowski, Alicja Sobaniec, Anna Gotz-Więckowska, Ewa Strauss

**Affiliations:** 1 Chair and Department of Ophthalmology, Poznan University of Medical Sciences, Poznan, Poland; 2 Institute of Human Genetics of the Polish Academy of Sciences, Strzeszynska 32, Poznan, 60-479 Poland; 3 Chair and Department of Neonatology, Poznan University of Medical Sciences, Poznan, Poland

**Keywords:** Retinopathy of prematurity, ROP, Nucleotide variants, Beta-2-adrenergic receptor, *ADRB2*, *AGTR1*, Genetics, Biomarkers, Molecular medicine, Risk factors

## Abstract

Retinopathy of prematurity (ROP) remains a leading cause of childhood blindness globally. The clinical progression of ROP exhibits notable similarities to infantile hemangioma (IH), suggesting shared risk factors and underlying mechanisms. This study aimed to investigate the influence of variants in genes postulated for IH—specifically, anthrax toxin receptor 1 (*ANTXR1*), beta-2-adrenergic receptor (*ADRB2*), Fms-related tyrosine kinase 4 receptor (*FLT4*), kinase insert domain receptor (*KDR*), and insulin-like growth factor 1 receptor (*IGF1R*)—on the development and severity of ROP. In our analysis of 210 infants born at a gestational age of less than 33 weeks, we identified the *ADRB2* rs1042714G variant allele as a significant risk factor for ROP, particularly its proliferative form. This risk was exacerbated by interactions with factors associated with neonatal respiratory failure, such as surfactant therapy, postnatal resuscitation, and mechanical ventilation, as well as the angiotensin II type 1 receptor variant (*AGTR1* rs5186A > C), previously linked to ROP risk in meta-analyses. Moreover, STRING protein-protein interaction analysis revealed that the ADRB2 protein interacts directly with a component of the vascular endothelial growth factor signaling pathway. These findings highlight potential pharmacological targets for ROP interventions, emphasizing the importance of understanding genetic contributions to this complex condition.

## Introduction

Retinopathy of prematurity (ROP) is a disease resulting from the disruption of retinal maturation and subsequent formation of abnormal blood vessels on the fundus of the eye in prematurely born infants, one of the most important causes of blindness worldwide^1^. The problem of vision loss due to ROP particularly affects children in low- and middle-income countries^2^. In these regions, ROP accounts for up to 40% of causes of vision loss^3^. In Poland, as well as in other European countries, ROP remains a major problem in pediatric ophthalmology. The percentage of preterm infants diagnosed with ROP varies between countries, but the proportion of infants requiring treatment has remained steady^4^. Improvements in perinatal and neonatal care standards are resulting in increased survival rates of the least mature newborns and an increase in the number of patients at risk for advanced stages of the disease. For instance, the observed incidence of the proliferative ROP has increased from 1.3% in 1996–2000 and 3.5% in 2001–2005 to values between 5.4% and 10% in 2008–2019^5–8^. Survival rates and clinical risk factors do not fully explain the observed trend^5^.

ROP has a difficult to predict course. It can progress rapidly to the most severe stages, leading to retinal detachment and vision loss, but also undergo spontaneous regression. Whole genome expression studies conducted in neonates with ROP confirm the key role of angiogenesis and hypoxia in the pathogenesis of the disease^9^. Vascular endothelial growth factor (VEGF) inhibitors have proven to be efficient pharmacotherapy in ROP, however there are serious concerns with systemic toxicity of this drugs due to non-selective VEGF blockade, as well as the risk of ROP re-activation after cessation of use^2^. Furthermore, there are no effective methods of preventing the disease. Therefore, there is a great need to develop methods for early detection of newborns most at risk. Identification of genetic markers for rapid and non-invasive diagnosis would not only allow us to better understand the cause of the disease, but also to develop and implement optimal treatment.

Genetic diagnosis is of growing interest in ROP research. A strong genetic predisposition to the disease was confirmed by Bizzarro et al.^10^ who analyzed 63 monozygotic and 137 dizygotic pairs of twins and found that genetic factors were responsible for 70.1% of the development of ROP. Similarly, in a study by Ortega-Molina et al.^11^, the heritability of ROP was 72.8%. Studies of hereditary retinopathies, such as familial exudative vitreoretinopathy and Norrie disease, have confirmed that variants in the *FZD4*, *LRP5*, *NDP* genes are responsible for a small percentage (3–12%) of advanced ROP^12–14^. However, none of the variants identified to date were present in the majority of patients with ROP and did not provide a sensitive and specific marker for the disease or its severe form. Major candidate genes include the gene for VEGF and their receptors, but there are also several other genes previously linked to ROP in both candidate gene^14–17^ and genome-wide^18^ association studies. Candidate genes for ROP also include those involved in the development of infantile hemangiomas (IH), due to their potential involvement in retinal neovascularization.

IHs are benign vascular tumors that occur more frequently in premature babies than in babies born at term. In a recent analysis of clinical databases, the occurrence of hemangiomas was associated with the incidence of ROP with an odds ratio (OR) of 3.13^19^. Many parallels in the clinical course of IH and ROP suggest common risk factors and pathogenesis. The two diseases share similar features (occurrence soon after birth, potential for spontaneous regression), molecular basis (hypoxia-induced angiogenesis plays a key role)^20–22^ and pharmacotherapy (both respond to treatment with the beta-blocker propranolol)^23,24^.

We hypothesized that single nucleotide variants (SNVs) in genes involved in the pathogenesis of IH also play a role in the development of ROP. We postulated the influence of variants in anthrax toxin receptor 1 (*ANTXR1*), beta-2-adrenergic receptor gene (*ADRB2*), Fms-related receptor tyrosine kinase 4 (*FLT4*, also known as vascular endothelial growth factor receptor precursor 3), insulin-like growth factor 1 receptor (*IGF1R*) and kinase insert domain receptor (*KDR*; also known as vascular endothelial growth factor receptor 2) genes^25–28^. Variants of these genes have previously been associated with the occurrence of IH (*ANTXR1*, *FLT4*, *KDR*) and malignancies (*IGF1R*), have the potential to modulate the pharmacotherapy effect of IH (*ADRB2*), or affect preterm birth and growth retardation (*ADRB2*, *IGF1R*), contributing more broadly to the pathogenesis of ROP. Our previous study, conducted on a smaller group of preterm infants, found an association between the *IGF1R* rs2229765 SNV and the incidence of severe intraventricular hemorrhage^29^, which also supports the role of these genes in complications of prematurity.

To test our hypothesis, we determined the associations between functional variants: *ANTXR1* rs119475040 (976G > A), *ADRB2* rs1042714 (79 C > G), *FLT4* rs34255532 (2860 C > T), *IGF1R* rs2229765 (3174G > A), *KDR* rs121917766, (3439 C > T), rs34231037 (1444T > C), and ROP in a group of 210 premature infants. Among them, *ADRB2*, *ANTXR1* and *FLT4* variants have not been previously studied in the context of ROP. To identify potential pathways and processes that may contribute to the pathogenesis of ROP, an in silico analysis was performed using the STRING database^30^, which included the genes studied and other genes previously associated with ROP. Gene-environment and gene-gene interactions were also evaluated based on the results.

## Results

### Characteristics of studied infants

The study included 210 premature infants simultaneously recruited into three groups of 70 infants: without ROP (the control group), with ROP undergoing spontaneous remission and with ROP requiring treatment, each containing 56% males (Table 1). The mean gestational age (GA) of the study group was 27.7 weeks (range: 23–32 weeks) and the mean birth weight (BW) was 1107.2 g (range: 490–2 340 g).

**Table 1:** Demographic and clinical parameters associated with retinopathy of prematurity (ROP).

	I No ROP *N* = 70	II ROP remission *N* = 70	III ROP progression *N* = 70	Statistical analysis *P* (χ^2^ or ANOVA)
Gestational age [wk.]				
Mean (SD)	29.2 (1.7)	27.6 (1.7)	26.3 (1.8)	< 0.0001
Range	24–32	23–30	23–32	
Body mass [g]				
Mean (SD)	1344.3 (339.2)	1057.6 (271.6)	919.8 (223.7)	< 0.0001
Range	640–2340	490–1800	500–1700	
Intrauterine hypotrophy; n (%)	3 (4.3)	9 (13.0)	2 (2.9)	0.037
Male sex; n (%)	39 (55.7)	39 (55.7)	39 (55.7)	0.975
Apgar 1; Median (Q1; Q3)	6 (4; 8)	4 (2; 6)	3 (1; 6)	< 0.0001
Apgar 5; Median (Q1; Q3)	7 (7; 9)	7 (6; 8)	7 (5; 7)	< 0.0001
No. of RCB transfusions; mean (SD)	1.84 (1.96)	4.1 (3.08)	6.5 (3.51)	< 0.0001
Risk factors at birth				
Ruptured fetal bladder; n (%)	19 (27.1)	21 (30.4)	22 (32.8)	0.766
Ruptured bladder time [d]; Mean (SD)	2.6 (5.8)	3.8 (9.9)	4.8 (13.6)	0.453
Delivery by caesarean section; n (%)	41 (58.6)	34 (49.3)	30 (44.1)	0.227
Parameters related to respiratory failure				
Surfactant treatment; n (%)	24 (34.8)	28 (41.2)	48 (70.6)	< 0.0001
Resuscitation; n (%)	49 (70.0)	60 (88.2)	66 (97.06)	< 0.0001
Mechanical ventilation; n (%)	39 (55.7)	50 (72.5)	68 (97.14)	< 0.0001
Mechanical ventilation time [d]; Mean (SD)	7.9 (12.4)	19 (18.7)	33.7 (21.2)	< 0.0001
Complications of prematurity; n (%)				
RDS	38 (54.3)	36 (52.2)	52 (76.5)	0.005
IVH	28 (40.0)	46 (66.7)	58 (85.3)	< 0.0001
NEC	10 (14.3)	18 (26.5)	24 (35.3)	0.017
BPD	9 (12.9)	31 (44.9)	48 (70.6)	< 0.0001
DWMI	1 (1.4)	8 (11.6)	9 (13.2)	0.028

*BPD*, bronchopulmonary dysplasia, *DWMI* diffuse white matter injury, *IVH* intraventricular hemorrhage, *RDS* respiratory distress syndrome, *NEC* necrotizing enterocolitis.

### Known risk factors and comorbidities

The presence of ROP and its advanced form was associated with GA (*P* < 0.001), BW (*P* < 0.0001), and intrauterine hypotrophy (*P* = 0.037) which seems to be a selective risk factor for ROP undergoing spontaneous remission. Additionally, there were significant associations between ROP development and Apgar scores at 1 min (*P* < 0.0001) and 5 min (*P* < 0.0001) after birth as well as with the number of red blood cell (RBC) transfusions (*P* < 0.0001). Significant associations were also observed between ROP development and parameters related to respiratory failure. The diagnosis of ROP was more frequent in patients who also developed other complications of prematurity, including respiratory distress syndrome (RDS), intraventricular hemorrhage (IVH), necrotizing enterocolitis (NEC), bronchopulmonary dysplasia (BPD), and diffuse white matter injury (DWMI).

### Genotypes and ROP in the studied population

The frequencies of all SNVs were consistent with Hardy-Weinberg equilibrium (HWE) (*P* > 0.05) (Table 2). For rare SNVs, no carriers of the *KDR* 3439T allele were found, while there were single heterozygous carriers of the *ANTXR1* 976 A and *FLT4* 2860T alleles. In the analysis of common SNVs, an association was observed between the *ADRB2* 79G allele and the development of ROP and its advanced form.

**Table 2:** The distribution of studied variants according to the presence and severity of retinopathy of prematurity (ROP).

Studied variant		I. No ROP *N* = 70	II ROP remission *N* = 70	III ROP progression *N* = 70	Statistical analysis: OR (95% CI); *P*			
					II vs. I	III vs. I	II + III vs. I	
Common SNV								
*ADRB2 79 C > G*	*CC*	25 (35.7)	17 (24.3)	11 (15.7)	1.0 (reference)	1.0 (reference)	1.0 (reference)	
	*CG*	31 (44.3)	35 (50)	31 (44.3)	1.7 (0.76–3.6); 0.205	2.3 (0.96–5.4); 0.06	1.3 (0.68–2.5); 0.423	
	*GG*	14 (20)	18 (25.7)	28 (40)	1.9 (0.75–4.8); 0.180	**4.5 (1.7–11.8); 0.002**	**2.9 (1.3–6.6); 0.009**	
	*MAF*	0.421	0.507	0.621	Dom: 1.7 (0.83–3.6); 0.142 Rec: 1.9 (0.75–4.8); 0.180	Dom: **3.0 (1.3–6.7); 0.008** Rec: **2.7 (1.3–5.7); 0.011**	Dom: **2.0 (1.1–3.9); 0.032** Rec: 2.0 (0.99–3.9); 0.054	
*IGF1R 3174G > A*	*AA*	22 (31.4)	18 (26.1)	23 (32.9)	1.0 (reference)	1.0 (reference)	1.0 (reference)	
	*GA*	33 (47.1)	35 (50.7)	32 (45.7)	ns	ns	ns	
	*GG*	15 (21.4)	16 (23.2)	15 (21.4)	ns	ns	ns	
	*MAF*	0.450	0.486	0.443	ns	ns	ns	
*KDR 1444T > C*	*TT*	62 (88.6)	64 (92.8)	66 (94.3)	1.0 (reference)	1.0 (reference)	1.0 (reference)	
	*TC*	8 (11.4)	5 (7.2)	3 (4.3)	ns	ns	ns	
	*CC*	0 (0)	0 (0)	1 (1.4)	ns	ns	ns	
	*MAF*	0.057	0.036	0.036	ns	ns	ns	
Rare SNV								
*ANTXR1 976G > A*	*GG*	70 (100)	70 (100)	69 (98.6)	1.0 (reference)	1.0 (reference)	1.0 (reference)	
	*GA*	0 (0)	0 (0)	1 (1.4)	ns	ns	ns	
	*MAF*	0.000	0.000	0.007	ns	ns	ns	
*FLT4 2860 C > T*	*CC*	69 (98.6)	70 (100)	70 (100)	1.0 (reference)	1.0 (reference)	1.0 (reference)	
	*CT*	1 (1.4)	0 (0)	0 (0)	ns	ns	ns	
	*MAF*	0.007	0.000	0.000	ns	ns	ns	
*KDR 3439 C > T*	*CC*	70 (100)	70 (100)	70 (100)	1.0 (reference)	1.0 (reference)	1.0 (reference)	
	*CT + TT*	0 (0)	0 (0)	0 (0)	na	na	na	
	*MAF*	0,000	0,000	0,000	na	na	na	

*[bold]*, statistically significant results; *Dom*, the dominant effect of the allele; *na*, not analyzed; *ns*, not significant; *Rec*, the recessive effect of the allele.

The *ADRB2* 79G allele was more common in ROP patients with spontaneous regression (0.507) and in those requiring treatment (0.621) than in infants without ROP (0.421). The effect of allele on ROP risk was allele-dependent. Compared to *ADRB2* 79CC homozygotes, 79CG heterozygotes had a 1.3-fold increased risk of developing any stage of ROP, while 79GG homozygotes had a 2.9-fold increased risk. The increased risk of advanced ROP associated with the presence of the genotypes above was 2.3-fold and 4.5-fold, for heterozygotes and homozygotes, respectively. Associations with ROP were statistically significant for the dominant effect of the 79G allele. Other studied variants were not associated with ROP (Table 2).

### Genotypes and risk factors of ROP and comorbidities

Table 3 shows the results of the analysis of the association between studied common SNVs, comorbidities of prematurity, extremely low gestational age (ELGA), and extremely low birth weight (ELBW). The *ADRB2* 79G allele was found to be statistically significantly associated with NEC and ELGA. In the study group of children born prematurely, the 79GG genotype was present almost 2 times more often in children with ELGA (*P* = 0.029), and its presence was further associated with a 2-fold higher risk of NEC (*P* = 0.039) The presence of the *IGF1R* 3174AA genotype was associated at the level of statistical trend with DWMI (OR = 1.9; *P* = 182; allele frequencies differed at *P* = 0.054).

**Table 3:** Distribution of common SNV according to the presence of complications of prematurity: RDS, IVH, PDA, NEC, BPD, ELGA, and ELBW.

Genotype	RDS		IVH		NEC		BPD		DWMI		GA		BW	
	No *N* = 81	Yes *N* = 126	No *N* = 75	Yes *N* = 132	No *N* = 154	Yes *N* = 52	No *N* = 119	Yes *N* = 88	No *N* = 189	Yes *N* = 19	≥ 28 wk. *N* = 122	< 28 wk. *N* = 87	≥ 1000 g *N* = 122	< 1000 g *N* = 87
*ADRB2 79 C > G*														
* CC*	22 (27.2)	30 (23.8)	22 (29.3)	30 (22.7)	43 (27.9)	9 (17.3)	33 (27.7)	19 (21.6)	50 (26.5)	2 (11.1)	34 (27.9)	18 (20.7)	33 (27.1)	19 (21.8)
* CG*	33 (40.7)	62 (49.2)	28 (37.3)	67 (50.8)	72 (46.8)	22 (42.3)	57 (47.9)	38 (43.2)	83 (43.9)	12 (66.7)	60 (49.2)	37 (42.5)	58 (47.5)	39 (44.8)
* GG*	26 (32.1)	34 (27)	25 (33.3)	35 (26.5)	39 (25.3)	21 (40.4)	29 (24.4)	31 (35.2)	56 (29.6)	4 (22.2)	28 (23.0)	32 (36.8)	31 (25.4)	29 (33.3)
* MAF*	0.525	0.516	0.52	0.519	**0.487**	**0.615** ^**a**^	0.483	0.568	0.516	0.556	**0.475**	**0.580** ^**b**^	0.492	0.557
*IGF1R 3174G > A*														
* AA*	20 (24.7)	43 (34.4)	18 (24.3)	45 (34.1)	45 (29.4)	18 (34.6)	30 (25.4)	33 (37.5)	55 (29.3)	8 (44.4)	37 (30.6)	26 (29.9)	32 (26.5)	31 (35.6)
* GA*	43 (53.1)	54 (43.2)	38 (51.4)	59 (44.7)	76 (49.7)	21 (40.4)	62 (52.5)	35 (39.8)	88 (46.8)	9 (50)	63 (52.1)	36 (41.4)	60 (49.6)	39 (44.8)
* GG*	18 (22.2)	28 (22.4)	18 (24.3)	28 (21.2)	32 (20.9)	13 (25)	26 (22)	20 (22.7)	45 (23.9)	1 (5.6)	21 (17.4)	25 (28.7)	29 (24)	17 (19.5)
* MAF*	0.488	0.440	0.500	0.436	0.458	0.452	0.483	0.426	**0.473**	**0.306** ^**c**^	0.434	0.494	0.488	0.420
*KDR 1444T > C*														
* TT*	74 (91.4)	115 (92)	66 (88)	123 (93.9)	140 (90.9)	49 (94.2)	107 (89.9)	82 (94.3)	172 (91.5)	17 (94.4)	109 (89.3)	82 (95.4)	110 (90.9)	81 (93.1)
* TC*	7 (8.6)	9 (7.2)	9 (12)	7 (5.3)	13 (8.4)	3 (5.8)	11 (9.2)	5 (5.8)	15 (8)	1 (5.6)	12 (9.8)	4 (4.7)	10 (8.3)	6 (6.9)
* CC*	0 (0.0)	1 (0.8)	0 (0.0)	1 (0.8)	1 (0.7)	0 (0.0)	1 (0.8)	0 (0.0)	1 (0.5)	0 (0.0)	1 (0.8)	0 (0.0)	1 (0.8)	0 (0.0)
* MAF*	0.043	0.044	0.06	0.034	0.049	0.029	0.055	0.029	0.045	0.028	0.057	0.023	0.05	0.034

*BPD* bronchopulmonary dysplasia, ELGA extremely low gestational age, *ELBW* extremely low birth weigh, *IVH* intraventricular hemorrhage, *DWMI* diffuse white matter injury, *RDS* respiratory distress syndrome, *ROP* retinopathy of prematurity, *NEC* necrotizing enterocolitis.

^a^NEC: frequency of the *ADRB2* 79G allele: OR 1.7 (95% CI 1.1–2.7); recessive model: OR = 2.0 (95% CI 1.0–3.9), *P* = 0.039.

^b^ELGA: frequency of the *ADRB2* 79G allele: OR 1.5 (95% CI 1.0–2.3), *P* = 0.034; recessive model: OR 1.95 (95% CI 1.1–3.6), *P* = 0.029.

^c^DWMI: frequency of the *IGF1R* 3174 A allele: OR 2.0 (95% CI 0.98–4.0), *P =* 0.054 (ns); recessive model: OR 1.9 (95% CI 0.53–5.2), *P =* 0.182 (ns).

### Gene x environment interactions

In the gene x environment (G x E) interaction analysis, we included ELGA, NEC as well as RDS and factors related to respiratory failure: surfactant treatment, resuscitation, and mechanical ventilation. These factors were selected based on the results of the analysis of associations (association with the *ADRB2* genotype, Table 2) and the results of previous expression studies indicating the presence of beta 2 adrenergic receptors in peripheral vessels and bronchi^31^. For ELGA or NEC we did not find any additive interaction concerning the occurrence of ROP (opposite results were observed; Supplementary Table S1a, b). In the case of RDS and respiratory failure-related factors, we observed that all of these factors exhibited a multiplicative interaction with the *ADRB2* genotype, particularly increasing the risk of ROP requiring treatment (Supplementary Table S1c–f). The effect of interaction on ROP requiring treatment was the strongest in the case of surfactant administration (OR for combined effect was 9.0; 95% CI (2.8–28.7), *P* = 0.0002; the Rothman synergy index (*S*) = 4.5) and mechanical ventilation (OR for combined effect was 43.0; 95% CI (2.4–785), *P* = 0.0001; *S* = 3.9). These effects were also present and significant when any type of ROP was considered.

### Gene x gene interactions

In the gene x gene (G x G) interaction analysis, we included the *ADRB2* gene variant and variants of other genes involved in the renin-angiotensin-aldosterone system (RAAS), that were studied in our previous study: the angiotensin-converting enzyme gene (*ACE*) I/D variant and angiotensin II receptor type 1 gene (*AGTR1*) rs5186A > C variant^32^. In the case of *ACE*, we assessed the effect on ROP occurrence, while in the case of *AGTR1*, the impact on ROP requiring treatment. Results are presented in (Supplementary Table S2). We observed a significant multiplicative interaction of the *AGTR1* rs5186C allele with the *ADRB2* rs10427142G (79G) allele. The individual effect of the *ADRB2* genotype increased the risk of ROP requiring treatment by 2.8-fold and *AGTR1* by 4-fold, while the combined effect of both increased the risk 17.5-fold (95% CI 3.0–102.6; *P* = 0.0008). The S-index was 3.4.

### Protein-protein interaction (PPI) network and module analysis

**Fig. 1:**
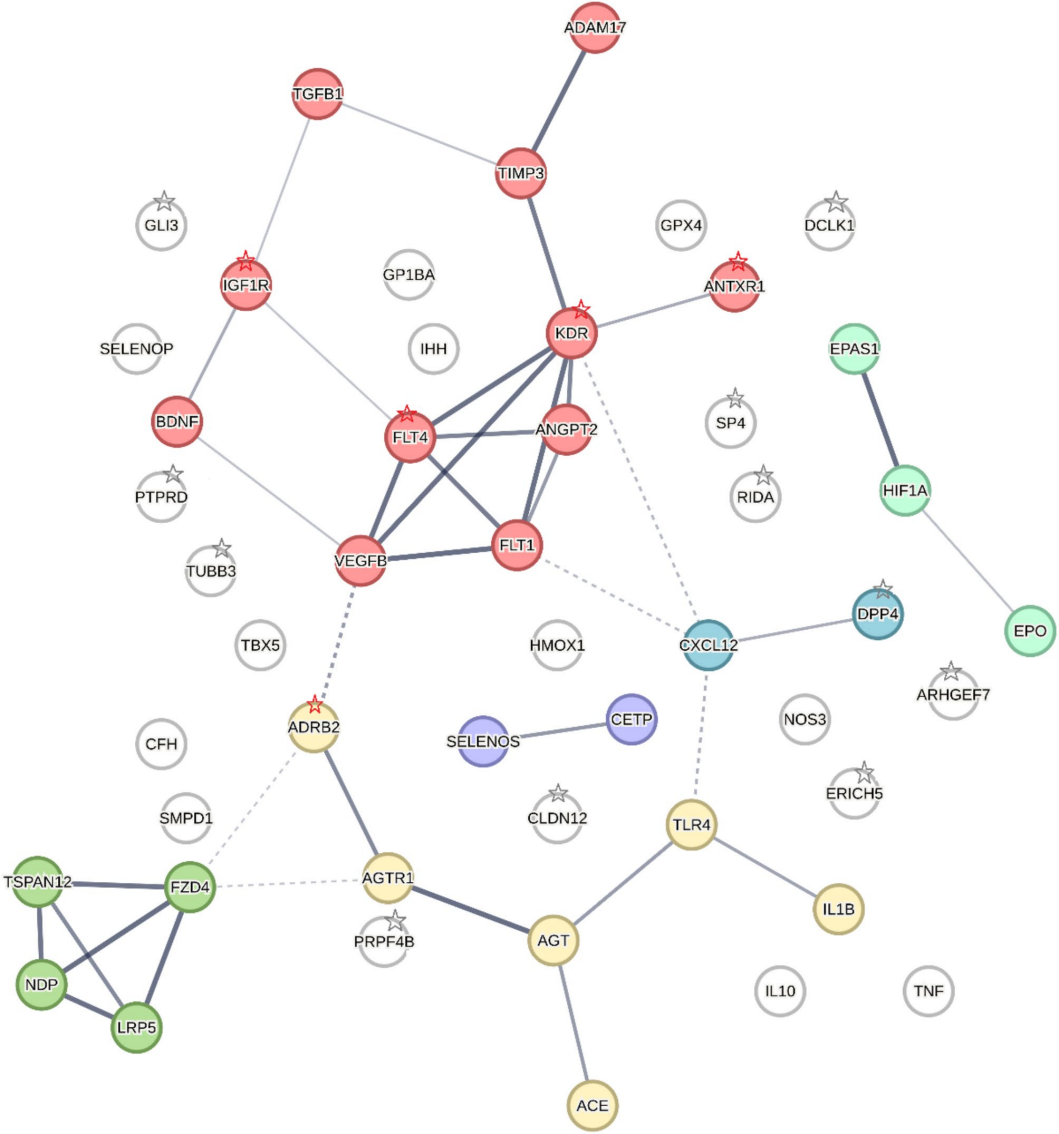
STRING PPI analyses. The nodes indicate the proteins and the edges indicate that the proteins are part of the physical complex. The thickness of the line refers to the degree of confidence prediction of the interaction. The color of the node denotes protein clusters: red—vascular endothelial growth factor signaling pathway; yellow—regulation of blood volume by renin-angiotensin; green—Norrin signaling pathway; violet—plasma protein particle; blue—*DPP4* and *CXCL12*; emerald—regulation of gene expression by hypoxia-inducible factor. Red asterisks indicate genes analyzed in this work, gray asterisks indicate genes identified in GWAS analysis. Other proteins refer to gene products studied in candidate gene association analyses.

The resulting PPI shown in (Fig. 1) confirms the availability of products of examined genes to create functional pathways and indicates that the directly linked proteins are part of the same physical protein complex. Six different pathways for studied proteins have been identified. The beta-2-adrenergic receptor was found to be active in the pathway that regulates blood volume by RAAS, including protein products of *AGTR1*,* AGT*,* ACE*,* TLR4*, and *IL1B*. All but one of the ROP genes identified with a significant *P*-value in GWAS, were located outside the clustered pathways. Only *DPP4* encoding dipeptidyl peptidase IV was clustered with the *CXCL12* encoding stromal cell-derived factor 1 in one pathway.

## Discussion

ROP represents one of the major challenges of pediatric ophthalmology because: (1) it has a difficult to predict course - so far it has not been possible to identify the factors that cause the progression of the disease to the most severe stages threatening retinal detachment and loss of vision; (2) it affects an increasing number of patients, due to the growing number of rescued premature infants with lower and lower birth weight and younger GA; (3) currently there are no known ways to prevent this disease. To achieve better monitoring and treatment, it seems important to understand the pathogenesis and etiology of this disease through genetic studies and correlate the information obtained with clinical data. To date, however, the contribution of specific genetic risk factors to ROP remains unclear.

In this study, we focused on three genes associated with the presence of IH and two with broader functions in the pathogenesis of ROP (Supplementary Table S3). Our analyses did not confirm the involvement of IH-related genes in the pathogenesis of ROP. They did, however, indicate the importance of the *ADRB2* gene, which may modulate the pharmacotherapy of IH. We found that the 79G allele was a risk factor for ROP, especially for the form requiring treatment. This allele was also associated with NEC and ELGA. The highest frequency of this allele was present in ROP requiring treatment (0.621) and NEC (0.615), followed by ELGA (0.580) and spontaneously resolving ROP (0.507). Its frequency in infants without ROP was 0.421, which corresponds to the frequency (0.436) in the healthy adult Polish population (blood donors)^33^. The effect of the *ADRB2* 79G allele on ROP was more substantial than in ELGA and NEC, which were significant only for homozygotes. Co-occurrence of NEC or ELGA with the *ADRB2* 79G allele did not further increase the risk of ROP, but factors associated with neonatal respiratory failure (including surfactant administration, postnatal resuscitation, and mechanical ventilation), as well as the presence of RDS (a multiplicative interaction) did. Interestingly, the same variant in a previous Polish population study showed an interaction with cigarette smoking increasing the risk of coronary artery disease^33^. In the present study, we also observed a suggestive trend for the *IGF1R* 3174 A allele to be associated with DWMI.

The *ADRB2* gene encodes the beta-2-adrenergic receptor. The rs1042714 (79 C > G) variant is a common SNV located in the protein-coding region. This variant causes an amino acid change in the protein (Gln27Glu), which has previously been associated with receptor desensitization^34^. This change in receptor activity ultimately leads to its reduced responsiveness to its ligands, which are catecholamines. The *ADRB2* rs1042714 variant has a relatively high prevalence in the Caucasian population and is one of the most common *ADRB2* variants with functional activity, making it a good candidate for association studies. It has been studied in the context of several common human diseases, including myocardial infarction, coronary artery disease, polycystic ovary syndrome, primary open-angle glaucoma, cancer, and preterm birth^35–38^.

Increasing evidence from experimental and clinical studies indicates that beta-adrenergic receptors allow retinal vessels to respond to hypoxia and may therefore further influence the development of ROP. Studies in a mouse model have shown that *VEGF*, *IGF1*, and *HIF1A* mRNA levels increase after hypoxia, but beta-adrenergic receptor blockade normalizes the levels of these transcripts^39^. In a subsequent clinical study, Filippi et al. tested the hypothesis that beta-blockade plays a role in inhibiting ROP. They used topical administration of the beta-blocker propranolol in eye drops. This drug is approved as a pharmacotherapy for cardiovascular conditions such as hypertension or tachycardia, but is also effective in the treatment of IH. The results indicated that propranolol may reduce the risk of progression from early-stage ROP to more severe forms^24^. They emphasize the safety of using propranolol drops, but point to its insufficient effectiveness in more advanced ROP and recommend conducting further clinical trials, both with propranolol and other types of drugs.

The effect of the *ADRB2* 79G gene variant on ROP was further strengthened by a multiplicative interaction with RDS and factors related to respiratory failure. The strongest interaction effect was observed for the advanced ROP phenotype and the combination of the *ADRB2* genotype with surfactant administration or mechanical ventilation (risk multiplied by a factor of 4). Respiratory failure is the main risk factor responsible for hypoxia in preterm infants, which additionally induces pathogenic pathways leading to neonatal oxidative stress and the development of ROP.

This specific interaction between the rs1042714 variant and the respiratory system may also be due to the shared function of beta-adrenergic receptors in the lungs and eyes. These receptors are expressed in tissues important for the cardiorespiratory system, including bronchial and arterial smooth muscle. Beta-adrenergic receptors enable bronchial and vascular dilation, including the retinal arteries^40^. Genetically determined changes in beta-adrenergic receptor activity may therefore affect both respiratory function and diseases associated with pathological neovascularization. Previous studies have shown associations between *ADRB2* gene variants and the development of congenital respiratory diseases in preterm infants, supporting a partial shared role of beta-adrenergic receptor pathways in the pathogenesis of respiratory disorders and ROP in preterm infants^41^.

PPI analysis revealed six major pathways involved in the pathogenesis of ROP, but only one of them, linking CXCL12 and DPP4, has a product of a gene identified in GWAS. In this cluster, CXCL12 is involved in hypoxia-induced angiogenesis, whereas DPP4 is an enzyme present in the fetal colon that disappears after birth. This analysis showed that a hypothesis-free study can be difficult to interpret, even if genes are identified. PPI analysis shows that ADRB2 is a protein in the RAAS, which also includes AGTR1, AGT, ACE, IL1B, and TLR4, that may directly interact with the vascular endothelial growth factor signaling pathway (via VEGFB)^42^. Furthermore, both ADRB2 and VEGFB have been mentioned in studies related to cardiovascular disease, vessel growth, and tumor angiogenesis^43,44^. They are also involved in the regulation of immune cells and play a role in the interaction with VEGF receptors. ADRB2 is one of the VEGF receptors that binds to VEGFB produced by nucleus pulposus cells, as shown by Tu et al.^45^. Because we have previously analyzed two other RASS components in ROP, *AGTR1* and ACE^32^, we performed a G x G interaction analysis for *ADRB* and *AGRT1* or *ACE* SNV (Supplementary Table S2). In this analysis, we observed that the cooccurrence of the *ADRB2* rs1042714G (79G) allele with the *AGRT1* rs5186C allele exceeds by a factor of 3.4 the effect expected from the sum of the separate impacts of these alleles (multiple interaction; Supplementary Table S2a). A similar interaction was not observed for the *ACE* gene variant (Supplementary Table S2b). PPI analysis confirms that *AGRT1* can directly interact with *ADRB2* (Fig. 1).

In our study, the *ADRB2* 79G allele was also associated with ELGA. This association may contribute to an increased risk of ROP, NEC, or IH in preterm infants and may partly represent a downstream effect of this allele on pregnancy complications. A study in a Korean population of 166 women showed an association between this allele and preterm birth^46^. The increased frequency of the 79G allele in children may therefore be a consequence of its frequency in mothers. Although our study did not analyze the maternal genotype, it should be noted that the frequency of the 79G allele observed in children with ELGA in this study (0.580) is 1.8 times higher than that observed in the healthy Polish population (0.436; OR 1.8; 95% CI (1.2–2.7); *P* = 0.006)^33^.

In addition, we observed a suggestive association between the *IGF1R* 3174G > A variant and DWMI. This disorder results from disruption of the myelination process of neurons in brain tissue and has potentially fatal consequences, preventing normal motor and cognitive development^47^. Prematurity is a risk factor for DWMI, increasing the likelihood of insufficient myelination of neurons at birth and rendering cells and surrounding glial tissue susceptible to oxidative stress^48^. The variant studied has previously been associated with melanoma, hypertension, as well as intrauterine growth retardation due to insulin-like growth factor I resistance and preterm birth^49^. It is a growth factor produced, among others, in the placenta that promotes fetal tissue development. Insufficient levels of this factor in utero have been correlated with fetal growth restriction, vascular abnormalities (including ROP and encephalopathy of prematurity), and BPD in preterm infants^50^. This factor is essential for brain tissue development because it controls glucose metabolism in neural tissue. Lower levels of insulin-like growth factor I are correlated with small brain volume in very preterm infants^51^.

STRING analysis has shown that by influencing VEGFB activity, it is possible to influence other proteins involved in the VEGF signaling pathway. Evaluation of gene variants that influence the vascular or angiogenic response in ROP, in particular further study of RAAS components, may provide valuable information on the pathogenesis of the disease and identify treatment strategies. ADRB2 acts as a natural regulator of vascular function and has physiological interactions that influence various cellular processes involved in the development of ROP.

A better understanding of the impact of individual variants on the course of ROP, i.e. their contribution to determining a mild course ending in spontaneous regression or a severe one leading to progression requiring treatment, which may end in retinal detachment and blindness, could help to identify a subgroup of high-risk newborns. Future research should address the importance of genetic variants in the design of new targeted treatments or prevention of ROP, the results of which will provide indications for intensified ophthalmic monitoring of at-risk newborns and provision of treatment at the most appropriate time, which is key to preventing progression. It also seems important to explore the possibility of personalizing drug therapy for ROP according to genetic predisposition. The role of *ADRB2* variants could be considered, since the variant analyzed in this study has previously been associated with inadequate response to heart disease treatment with the beta-blocker metoprolol^52^.

### Limitations

Several limitations of our study should be highlighted. The first limitation is the lack of follow-up of infants tested for ROP for symptoms of IH, because IH usually does not occur immediately after birth. Second, it would be interesting to analyze the influence of variants in the studied genes, especially *ADRB2* and *IGFR1*, on intrauterine growth restriction, but the number of cases with intrauterine hypotrophy identified in this study (*n* = 14, Table 1) was too small to perform such an analysis. This area is a promising direction for further research. Since the mothers of preterm infants were not genotyped, the third drawback of this study is the lack of information regarding the role of the studied variants in the occurrence of pregnancy complications leading to preterm birth. The fourth limitation is the relatively small sample size in the genetic analyses. However, post-hoc statistical power analysis confirmed that the obtained results for most analyses were highly reliable. For example, when assessing the effect of the *ADRB2* 79G allele on the occurrence of ROP requiring treatment, statistical power analysis showed power of 97, 75, and 92% for recessive, dominant, and additive models. For the G x E interaction, the statistical power of the interaction between *ADRB2* and mechanical ventilation was 75%, whereas for *ADRB2* and RDS, surfactant administration, or resuscitation, it ranged from 60 to 65%. For the G x G interaction, the statistical power of the observed interaction between *ADRB2* and *AGTR1* was 84%. The last limitation is that we cannot exclude the possibility that the identified G x E effects are overestimated in our study, as the need for surfactant administration and mechanical ventilation, along with low GA and oxygen therapy, are known and clinically confirmed risk factors for ROP. The identified variants may serve as markers of predisposition; however, further studies are required to confirm their role in disease progression and phenotype severity.

## Conclusion

In conclusion, we found that a variant of the gene involved in IH formation, specifically the *ADRB2* rs1042714G allele is a risk factor for the development of ROP and the presence of its advanced stages. The impact of this gene variant on ROP may be more substantial in newborns with respiratory failure and in carriers of the *AGTR1* rs5186C allele. The observed effects require replication in a larger sample and other populations.

## Methods

### Study population

The study included a population of 210 preterm infants who met the criteria for ROP screening as defined by the consensus of the Polish Neonatologists and Pediatric Ophthalmology Section. These criteria included infants with a gestational age (GA) of < 33 weeks, and a birth weight (BW) of ≤ 1800 g^53^. Infants were included in three equally sized and gender-matched subgroups: without ROP (the control group), ROP with spontaneous remission and ROP requiring treatment (Table 1). Study group was collected between 2009 and 2016 from the Neonatal Intensive Care Unit (NICU) at the Clinical Hospital of Gynecology and Obstetrics at Poznan University of Medical Sciences. This hospital is a tertiary referral hospital and the largest obstetrics center in Poland, with an annual birth rate exceeding 7000. All newborns included in the study were of Caucasian origin. Exclusion criteria for the study comprised infants born from multiple pregnancies and those with chromosomal abnormalities.

### Clinical features and ROP management

Clinical factors that may impact the development of ROP were collected from the patient’s medical records. These factors included gender, GA (in weeks), BW (in grams), Apgar score at the 1st and 5th minute, mode of delivery (vaginal birth or cesarean section), presence of ruptured fetal bladder, intrauterine hypotrophy (defined as fetal birth weight below the 10th percentile for GA) and parameters related to respiratory failure such as surfactant treatment, resuscitation, or mechanical ventilation. GA ≤ 28 weeks was defined as extremely low GA (ELGA), while BW ≤ 1800 g. as extremely low BW (ELBW). Additionally, the presence of other complications of prematurity, including bronchopulmonary dysplasia (BPD), respiratory distress syndrome (RDS), intraventricular hemorrhage (IVH), necrotizing enterocolitis (NEC), and diffuse white matter injury (DWMI), was recorded. The diagnostic criteria for RDS, NEC, IVH, BPD, and NEC have been described in previous studies^54^, while DWMI was diagnosed based on abnormal ultrasound or MRI findings.

The premature newborns included in the study underwent regular ROP screening, which involved a series of eye fundus examinations using binocular indirect ophthalmoscopy. The examinations were conducted under topical anesthesia (proxymetacaine) and after pupil dilation (tropicamide 1% and phenylephrine 2.5% drops). The initial examination was performed in the 4th week of chronological age, with subsequent follow-up examinations scheduled every 7–10 days, depending on the condition of the eyes. In cases where ROP was diagnosed, the fundus lesions were classified according to the International Classification of Retinopathy of Prematurity (ICROP). Treatment was administered based on the guidelines outlined in the Early Treatment for Retinopathy of Prematurity. Patients with any stage of ROP in zone I with plus disease, stage 3 ROP with no plus disease in zone I, or stage 2 or 3 ROP with plus disease in zone II were eligible for treatment^55^. The treatment options included laser photocoagulation of the peripheral avascular retina or intravitreal administration of an anti-VEGF antibody (ranibizumab) within 72 h of diagnosis. The ROP screening continued until the vascularization reached zone III or until signs of ROP regression were observed in at least two consecutive examinations.

### Genotyping

For DNA isolation 1 ml of blood from peripheral vein were taken from the patient’s during routine testing. Alternatively, cheek swabs were taken (in duplicate). Samples were frozen and delivered to the Institute of Human Genetics of the Polish Academy of Sciences, where genomic DNA was isolated and the genetic analysis was performed. Genomic DNA from buccal swabs was extracted using the innuPREP DNA Kit (Analytik Jena AG, Jena, Germany) and from circulating blood lymphocytes using the QIAamp DNA Kit (Qiagen GmbH, Hilden, Germany) following the manufacturer’s instructions. The criteria for the selection of candidate genes in this study were their potential involvement in the pathogenesis of ROP. Three common variants: *ANTXR1* rs119475040, *IGF1R* rs2229765, and *KDR* rs34231037, and three rare variants (< 5%) *ADRB2* rs1042714; *FLT4* rs34255532, and *KDR* rs121917766 were analyzed. The single nucleotide variants were determined using quantitative polymerase chain reaction (qPCR) with commercially available pre-designed genotyping assays based on TaqMan probes (Thermo Fisher Scientific, C_154335568_10; C_137540_1; C_25612213_20; C_2084765_20; C_62629284_10; C_170050247_10) were used and the ABI 7900HT Fast Real-Time PCR System (Life Technologies, Carlsbad, CA) employed. Details of the genetic variants and tests used for genotyping are shown in (Supplementary Table S4).

### Construction of PPI network and module analysis

STRING (version 12.0)^30^ was used to evaluate the potential protein-protein relationships among protein products of analyzed genes, selected based on their involvement in pathogenic pathways of IH (*ADRB2*, *ANTXR1*, *FLT4*, *IGF1R*,* KDR)*, and protein products of other genes previously associated with ROP, selected based on literature data^14,17,18^; (Supplementary Table S5). The PPI network was constructed using default parameters (a medium confidence score threshold of > 0.4). The k-means algorithm was used to cluster the proteins with the desired/defined number of 6 clusters.

### Statistical analysis

Testing for HWE was conducted using a *χ*^2^ test available at the following URL: https://ihg.helmholtz-muenchen.de/cgi-bin/hw/hwa1.pl (accessed on 1 January 2023). To evaluate the individual effects of the studied factors on the onset and progression of ROP, a *χ*^2^ or ANOVA test was performed. Factors showing deviations from a normal distribution in the Kolmogorov-Smirnov test were examined after being subjected to logarithmic transformation. In the univariate analyses, qualitative variables were examined using the *χ*^2^ test or Fisher’s test, while quantitative variables were evaluated using the *t*-test or Mann-Whitney U test. ORs with 95% CIs were calculated for the genotypes, and the impact of MAF of the studied variants was assessed in these analyses. The recessive and dominant models of interactions were tested, along with the effect of combinations of genotypes. G x E interactions were assessed using the method described by Botto and Khoury^56^, which is based on the use of a multivariate table with a two-by-four layout. The assessment was performed using univariate analyses (*χ*^2^ or Fisher’s test). Expected ORs from additive models were calculated. The Rothman synergy index (S), which indicates a deviation from the additive model of interactions, was also determined. The interpretation of the coefficient values is as follows: *S* = 1 indicates no interaction; *S* < 1 indicates a relative decrease; and *S* > 1 indicates an increase in the strength of the interactions between the two factors. The same method was adopted for the analysis of G x G interactions.

Association analysis was performed for both ROP and ROP requiring treatment. All tests were two-sided, and a significance level of *P* < 0.05 was considered statistically significant. Except for PPI analysis, STATISTICA version 10.0 and GraphPad Prism version 6.04 software were used to perform statistical analyses. Post hoc power analysis for associations was conducted using Quanto software.

### Ethics statement

All procedures carried out on human participants in this study were in accordance with the ethical standards of the institutional and/or national research committee, and with the 1964 Helsinki Declaration and its later amendments (or comparable ethical standards). The study was approved by the Bioethics Committee of Poznan University of Medical Sciences (no. 1140/05, 1117/18, 45/22 and 126/22). Written prior-informed consent was obtained from the parents or guardians of the patients.

## Electronic supplementary material

Below is the link to the electronic supplementary material.

Supplementary Material 1

## Data Availability

All data generated or analyzed during this study are included in the published article (and its Supplementary Information files). Further information is available from the corresponding author upon reasonable request.
