# Supplementary material for: Association of the ADRB2 rs1042714 variant with retinopathy of prematurity highlights the importance of the renin-angiotensin-aldosterone system
Source: Sci Rep. 2025 Apr 2;15:11232. doi: 10.1038/s41598-025-95055-1 (PMC11965322; doi:10.1038/s41598-025-95055-1)
Supplement: Supplementary file 1 — Supplementary Material 1 [file 41598_2025_95055_MOESM1_ESM.pdf]

Supplementary materials

Association of the *ADRB2* rs1042714 variant with retinopathy of prematurity highlights the importance of the renin-angiotensin-aldosterone system

Anna Chmielarz-Czarnocińska[1], Anna Durska[2*^] Bartosz Skulimowski[1^]; Alicja Sobaniec[3], Anna Gotz-Więckowska[1], Ewa Strauss[2*]

[1] Chair and Department of Ophthalmology, Poznan University of Medical

Sciences, Poznan, Poland

[2] Institute of Human Genetics of The Polish Academy of Sciences, Poznan, Poland

[3] Chair and Department of Neonatology, Poznan University of Medical Sciences, Poznan, Poland

^-equal contribution (A.D) and (B.S.)

*-corresponding author

Ewa Strauss

The Institute of Human Genetics, Polish Academy of Sciences,

Strzeszynska 32, 60-479 Poznan, Poland

Electronic address: strauss@man.poznan.pl

ORCID 0000-0001-8275-3126

Supplementary Table S1. The effect of gene-environment interaction between the *ADRB2* genotype and the extremely low gestational age (ELGA) (a), necrotizing enterocolitis (NEC) (b), parameters related to respiratory failure: surfactant administration (c), resuscitation at birth (d), mechanical ventilation (e) respiratory distress syndrome (RDS) (f) on the incidence and severity of retinopathy of prematurity (ROP). Odds Ratios (ORs) expected from the additive model and the Rothman synergy indexes (*S*-values) were calculated.

a)

Risk factors		Incidence and outcome of ROP			Statistical analysis OR (95%CI); *P*	
ADRB2 rs1042714	ELGA	I No ROP	II ROP not requiring treatmen	III ROP requiring treatment	II vs. I	III vs. I
CC	≥28 wk.	24 (34.3)	7 (4.3)	3 (10.1)	reference	reference
CC	<28 wk.	1 (1.4)	9 (11.4)	8 (13)	30.9 (3.3 - 287.4); 0.0006	64 (5.8 - 706.2); < 0.0001
CG+GG	≥28 wk.	39 (55.7)	36 (18.6)	13 (52.2)	3.2 (1.2 - 8.2); 0.027	2.7 (0.69 - 10.3); 0.245
CG+GG	**<28 wk.**	**6 (8.6)**	**17 (65.7)**	**46 (24.6)**	**9.7 (2.8 - 34.1); 0.0005**	**61.3 (14.1 - 267.2); < 0.0001**
		OR expected from the additive model[a], S			*33;* ***0.3*↓**	*65.7;* ***0.9*↓**

a-Expected OR= OR1 $_{\text{Observed}}$ (CC and <28 wk.) + OR2 $_{\text{Observed}}$ (CG+GG and ≥28 wk.) -1

b)

Risk factors		Incidence and outcome of ROP			Statistical analysis OR (95%CI); *P*	
ADRB2 rs1042714	NEC	I No ROP	II ROP not requiring treatmen	III ROP requiring treatment	II vs. I	III vs. I
CC	No	24 (34.3)	11 (16.2)	8 (11.8)	reference	reference
CC	Yes	1 (1.4)	5 (7.4)	3 (4.4)	10.9 (1.1 -104.9); 0.050	9.0 (0.82 - 99.3); 0.141
CG+GG	No	36 (51.4)	39 (57.4)	36 (52.9)	2.4 (1.0 - 5.5); 0.070	3.0 (1.2 - 7.6); 0.030
CG+GG	**Yes**	**9 (12.9)**	**13 (19.1)**	**21 (30.9)**	**3.2 (1.0 - 9.6); 0.075**	**7.0 (2.3 - 21.4); 0.001**
		OR expected from the additive model[a], S			*12.3;* ***0.2*↓**	*11.0;* ***0.6*↓**

a-Expected OR= OR1 $_{\text{Observed}}$ (CC and NEC) + OR2 $_{\text{Observed}}$ (CG+GG and no-NEC) -1

c)

Risk factors		Incidence and outcome of ROP			Statistical analysis OR (95%CI); *P*	
ADRB2 rs1042714	Surfactant	I No ROP	II ROP not requiring treatmen	III ROP requiring treatment	II vs. I	III vs. I
CC	No	16 (23.2)	9 (13.2)	5 (7.4)	reference	reference
CC	Yes	9 (13)	7 (10.3)	6 (8.8)	1.4 (0.38 - 5.0); 0.746	2.1 (0.50 - 9.0); 0.465
CG+GG	No	29 (42)	31 (45.6)	15 (22.1)	1.9 (0.73 - 5.0); 0.236	1.7 (0.51 - 5.4); 0.567
CG+GG	**Yes**	**15 (21.7)**	**21 (30.9)**	**42 (61.8)**	**2.5 (0.87 - 7.1); 0.075**	**9.0 (2.8 - 28.7); 0.0002**
		OR expected from the additive model[a], S			*2.3;* ***1.2*↑**	*2.8;* ***4.5*↑**

a-Expected OR= OR1 $_{\text{Observed}}$ (CC and Surfactant) + OR2 $_{\text{Observed}}$ (CG+GG and without surfactant) -1

Statistical analysis OR (95%CI); *P* groups: II+III vs I CG+GG and Surfactant: 4.8 (1.9-12.0); 0.0008 (OR exp = 2.5; S = 2.6)

d)

Risk factors		Incidence and outcome of ROP			Statistical analysis OR (95%CI); *P*	
ADRB2 rs1042714	Resuscitation at birth	I No ROP	II ROP not requiring treatmen	III ROP requiring treatment	II vs. I	III vs. I
CC	No	5 (7.1)	2 (2.9)	0 (0.0)	reference	reference
CC	Yes	20 (28.6)	14 (20.6)	11 (16.2)	1.75 (0.30-10.3); 0.684	6.2 (0.31-122); 0.295
CG+GG	No	16 (22.9)	6 (8.8)	2 (2.9)	0.94 (0.14-6.2); 1.00	1.7 (0.07-40.3); 1.00
CG+GG	**Yes**	**29 (41.4)**	**46 (67.6)**	**55 (80.9)**	**3.97 (0.72– 21.8); 0.120**	**20.7 (1.1 - 379); 0.007**
		OR expected from the additive model[a], S			*1.7;* ***4.3*****↑**	*6.8;* ***3.0*****↑**

a-Expected OR= OR1 Observed (CC and Resuscitation) + OR2 Observed (CG+GG and without Resuscitation) -1

Statistical analysis OR (95%CI); *P* groups: II+III vs I CG+GG and Res.: 8.7 (1.6-47.3); 0.01 (OR exp = 3.5; S = 3.1)

e)

Risk factors		Incidence and outcome of ROP			Statistical analysis OR (95%CI); *P*	
ADRB2	Mechanical ventilation	I No ROP	II ROP not requiring treatmen	III ROP requiring treatment	II vs. I	III vs. I
CC	No	8 (11.4)	2 (2.9)	0 (0)	reference	reference
CC	Yes	17 (24.3)	14 (20.3)	11 (15.7)	3.3 (0.60 - 18); 0.265	11 (0.59 - 213); 0.076
CG+GG	No	23 (32.9)	17 (24.6)	2 (2.9)	3 (0.56 - 16); 0.282	1.8 (0.079 - 42); 1
CG+GG	**Yes**	**22 (31.4)**	**36 (52.2)**	**57 (81.4)**	**6.5 (1.3 - 34); 0.018**	**43.4 (2.4 - 785); 0.0001**
		OR expected from the additive model[a], S			*5.3;* ***1.3*****↑**	*11.6;* ***3.9*****↑**

a-Expected OR= OR1 Observed (CC and Res) + OR2 Observed (CG+GG and no-Res) -1; b- *The Rothman synergy index (S)*

Statistical analysis OR (95%CI); *P* groups: II+III vs I CG+GG and Res.: 16.9 (3.4-85.0); 0.0002 (OR exp = 8.2; S = 2.2)

f)

Risk factors		Incidence and outcome of ROP			Statistical analysis OR (95%CI); *P*	
ADRB2 rs1042714	RDS	I No ROP	II ROP not requiring treatmen	III ROP requiring treatment	II vs. I	III vs. I
CC	No	11 (15.7)	8 (11.6)	3 (4.4)	reference	reference
CC	Yes	14 (20.0)	8 (11.6)	8 (11.8)	0.79 (0.22-2.8); 0.757	2.1 (0.45-9.8); 0.467
CG+GG	No	21 (30.0)	25 (36.2)	13 (19.1)	1.64 (0.56-4.8); 0.422	2.3 (0.53-9.7); 0.327
CG+GG	**Yes**	**24 (34.3)**	**28 (40.6)**	**44 (64.7)**	**1.6 (0.55– 4.6); 0.381**	**6.7 (1.7 – 26.5); 0.006**
		OR expected from the additive model[a], S			*1.4;* ***1.4*****↑**	*3.4;* ***2.4*****↑**

a-Expected OR= OR1 Observed (CC and RDS) + OR2 Observed (CG+GG and no-RDS) -1

Statistical analysis OR (95%CI); *P* groups: II+III vs I CG+GG and RDS: 3.0 (1.2-7.8); 0.036 (OR exp = 2.0; S = 2.1)

Supplementary Table S2. The effect of gene-gene interaction between the *ADRB2* genotype and *ACE* (a) or *AGTR1* (b) or genotypes on the incidence and severity of retinopathy of prematurity (ROP).

a)

Risk factors		Incidence and outcome of ROP			Statistical analysis OR (95%CI); *P*
ADRB2 rs1042714	*ACE* *I/D*	I No ROP	II ROP not requiring treatmen	III ROP requiring treatment	III +II vs. I (ROP occurrence)
CC	II	5 (7.2)	2 (2.9)	0 (0.0)	reference
CC	ID+DD	20 (28.6)	15 (21.4)	11 (15.7)	3.2 (0.57-18.5); 0.234
CG+GG	II	10 (14.3)	11 (15.7)	17 (24.3)	7.0 (1.2-42.0); 0.032
CG+GG	ID+DD	**34 (48.6)**	**42 (60.0)**	**42 (60.0)**	**6.2 (1.1– 33.4); 0.030**
		OR expected from the additive model[a], S			*9.3;* ***0.6*↓**

a-Expected OR= OR1 $_{\text{Observed}}$ (CC and ID+DD) + OR2 $_{\text{Observed}}$ (CG+GG and II) -1

b)

Risk factors		Incidence and outcome of ROP			Statistical analysis OR (95%CI); *P*
ADRB rs10427142	*AGTR1* *rs5186*	I No ROP	II ROP not requiring treatmen	III ROP requiring treatment	III vs. I + II (ROP requiring treatment)
CC	AA	4 (20.9)	8 (12.5)	1 (1.5)	reference
CC	AC+CC	9 (13.4)	9 (14.1)	10 (14.7)	4.0 (0.85-18.8); 0.087
CG+GG	AA	22 (32.8)	32 (50.0)	27 (39.7)	2.8 (1.2-6.8); 0.022
CG+GG	AC+CC	**22 (32.8)**	**15 (23.4)**	**30 (44.1)**	**17.5 (3.0– 102.6); 0.0008**
		OR expected from the additive model[a], S			*5.8;* ***3.4*↑**

a-Expected OR= OR1 $_{\text{Observed}}$ (CC and AC+CC) + OR2 $_{\text{Observed}}$ (CG+GG and AA) -1

Supplementary Table S3. Genes and variants selected for the association study with clinical significance (based on: NCBI Reference SNP (rs) Report and PubMed).

Gene name	Variant	Clinical Significance (ClinVar)	Associated Disease / Feature	Description and Reference
ADRB2:<br>beta-2 adrenergic receptor gene	rs1042714	Allele: G<br><br>Benign	Infantile hemanglioma pharmacotherapy	Association between genetic polymorphisms of beta-2 adrenergic receptor and the heart rate **response of β-blockers** -2019<br>https://pubmed.ncbi.nlm.nih.gov/31090079/
			Preterm delivery (maternal factor)	Associations between genetic polymorphisms of beta-2 adrenergic receptor and **preterm delivery** in Korean women – 2013<br>https://pubmed.ncbi.nlm.nih.gov/22985077/
ANTXR1:<br>anthrax toxin receptor 1 isoform 1 precursor gene	rs119475040	Allele: A<br><br>Risk-Factor	Susceptibility to Capillary infantile hemangioma	Suppressed NFAT-dependent VEGFR1 expression and constitutive VEGFR2 signaling in **infantile hemangioma** – 2008<br>https://pubmed.ncbi.nlm.nih.gov/18931684/
FLT4:<br>fms related receptor tyrosine kinase 4 gene	rs34255532	Allele: A<br><br>Pathogenic	Susceptibility to Capillary infantile hemangioma	Somatic mutation of vascular endothelial growth factor receptors in **juvenile hemangioma –** 2022<br>https://pubmed.ncbi.nlm.nih.gov/11807987/
IGF1R:<br>Insulin-like growth factor 1 receptor gene	rs2229765	Allele: A<br><br>Benign	Susceptibility to melanoma and hypertension	A Possible Link of Genetic Variations in ER/IGF1R Pathway and Risk of **Melanoma** 2020<br>https://pubmed.ncbi.nlm.nih.gov/32150843/<br>Insulin-Like Growth Factor-1 and Receptor Contribute Genetic Susceptibility to **Hypertension** in a Han Chinese Population 2018<br>https://pubmed.ncbi.nlm.nih.gov/29126188/
			Growth delay due to insulin-like growth factor I resistance (maternal factor)	Maternal IGF1 and IGF1R polymorphisms and the risk of **spontaneous preterm birth** – 2017 https://pubmed.ncbi.nlm.nih.gov/28213921/
KDR:<br>kinase insert domain receptor gene	rs121917766	Allele: A<br><br>Pathogenic	Susceptibility to Capillary infantile hemangioma	Somatic mutation of vascular endothelial growth factor receptors in **juvenile hemangioma** – 2022 https://pubmed.ncbi.nlm.nih.gov/11807987/
	rs34231037	Allele: G<br><br>Risk-Factor	Susceptibility to Capillary infantile hemangioma	Somatic mutation of vascular endothelial growth factor receptors in **juvenile hemangioma** – 2022 https://pubmed.ncbi.nlm.nih.gov/11807987/<br>Identification of a variant in KDR associated with serum VEGFR2 and **pharmacodynamics** of Pazopanib. – 2015<br>https://pubmed.ncbi.nlm.nih.gov/25411163/

Supplementary Table S4. Methodology for evaluating the studied variants.

Variant	Fluorescent marker of the probe	Context sequence	Thermo Fisher Scientific Assay ID
ADRB2 rs1042714	VIC/FAM	TGCGCCGGACCACGACGTCACGCAG[**C/G**] AAAGGGACGAGGTGTGGGTGGTGGG	C___2084765_20
ANTXR1 rs119475040	VIC/FAM	GTCTGACGGTTCCATCCTGGCCATC[**A/G**] CCCTGCTGATCCTGTTCCTGCTCCT	C_154335568_10
FLT4 rs34255532	VIC/FAM	GCGCGGAAGCGTCCGCGCTGCTCGG[**A/G**] AGACTTCTCCTGCGGATGCACGAAG	C__62629284_10
I*GF1R* rs2229765	VIC/FAM	TGAACGAGGCCGCAAGCATGCGTGA[**A/G**] AGGATTGAGTTTCTCAACGAAGCTT	C____137540_1_
KDR rs121917766	VIC/FAM	TCTGAAAACGTGGGTCTCTGACTGG[**G/A**] CTCCCCGTGCCAGCAGTCCAGCATG	C_170050247_10
KDR rs34231037	VIC/FAM	TCCTCCACACTTCTCCATTCTTCAC[**A/G**] AGGGTATGGGTTTGTCACTGAGACA	C__25612213_20

qPCR conditions

Steps	Temperature (°C)	Duration	Cycles
Initial Denaturation	95 °C	12 min	1
Denaturation	95 °C	20 s	1
Annealing/Extension	60 °C	60 s	40

Supplementary Table S5. Genes selected for the STRING PPI analysis.

Gene name	HGNC Approved Gene Symbol	Gene ID	Cytogenetic location
Angiotensin I-converting enzyme	*ACE*	106180	17q23.3
Tumor necrosis factor-alpha converting enzyme	*ADAM17*	* 603639	2p25.1
Angiotensinogen	*AGT*	106150	1q42.2
Angiotensin II receptor, type 1	*AGTR1*	* 106165	3q24
Angiopoietin 2	*ANGPT2*	* 601922	8p23.1
Brain-derived neurotrophic factor	*BDNF*	* 113505	11p14.1
Cholesteryl ester transfer protein	*CETP*	* 118470	16q13
Complement factor H	*CFH*	* 134370	1q31.3
Stromal cell-derived factor 1	*CXCL12*	* 600835	10q11.21
Endothelial pas domain protein 1	*EPAS1*	* 603349	2p21
Erythropoietin	*EPO*	* 133170	7q22.1
Fms-related tyrosine kinase 1/vascular endothelial growth factor receptor 1	*FLT1 / VEGFR1*	* 165070	13q12.3
Frizzled class receptor 4	*FZD4*	* 604579	11q14.2
Glycoprotein ib	*GP1BA*	* 606672	17p13.2
Hypoxia-inducible factor 1, alpha subunit	*HIF1A*	* 603348	14q23.2
Heme oxygenase 1	*HMOX1*	* 141250	22q12.3
Insulin-like growth factor I receptor	*IGF1R*	* 147370	15q26.3
Indian hedgehog signaling molecule	*IHH*	* 600726	2q35
Interleukin 10 / cytokine synthesis inhibitory factor	*IL10*	* 124092	1q32.1
Interleukin 1-beta	*IL1B*	* 147720	2q14.1
Kinase insert domain receptor	*KDR*	* 191306	4q12
Low density lipoprotein receptor-related protein 5	*LRP5*	* 603506	11q13.2
Norrin cystine knot growth factor NDP	*NDP*	* 300658	Xp11.3
Nitric oxide synthase 3	*NOS3*	163729	7q36.1
Sphingomyelin phosphodiesterase 1	*SMPD1*	* 607608	11p15.4
T-box transcription factor 5	*TBX5*	* 601620	12q24.21
Transforming growth factor, beta-1	*TGFB1*	* 190180	19q13.2
Tissue inhibitor of metalloproteinase 3	*TIMP3*	* 188826	22q12.3
Toll-like receptor 4	*TLR4*	* 603030	9q33.1

Tumor necrosis factor	*TNF*	* 191160	6p21.33
Tetraspanin 12	*TSPAN12*	* 613138	7q31.31
Vascular endothelial growth factor B	*VEGFB*	* 601398	11q13.1
**Reference:** Swan R, Kim SJ, Campbell JP, Paul Chan RV, Sonmez K, Taylor KD, Li X, Chen YI, Rotter JI, Simmons C, Chiang MF; Imaging and Informatics in ROP Research Consortium. The genetics of retinopathy of prematurity: a model for neovascular retinal disease. Ophthalmol Retina. **2018**;2(9):949-962. doi: 10.1016/j.oret.2018.01.016. PMID: 30250936; PMCID: PMC6150458.			
Glutathione peroxidase 4	*GPX4*	* 138322	19p13.3
Selenoprotein P	*SELENOP*	* 601484	5p12
Selenoprotein S	*SELENOS*	* 607918	15q26.3
**Reference:** Strauss E, Januszkiewicz-Lewandowska D, Sobaniec A, Gotz-Więckowska A. *SELENOP* rs3877899 Variant Affects the Risk of Developing Advanced Stages of Retinopathy of Prematurity (ROP). Int J Mol Sci. **2023**;24(8):7570. doi: 10.3390/ijms24087570. PMID: 37108730; PMCID: PMC10145309			
Rho guanine nucleotide exchange factor 7	*ARHGEF7*	* 605477	13q34
Claudin 12	*CLDN12*	* 611232	7q21.13
Doublecortin-like kinase 1	*DCLK1*	* 604742	13q13.3
Dipeptidyl peptidase IV	*DPP4*	* 102720	2q24.2
Glutamate-rich protein 5	*ERICH5*	* 620895	8q22.2
Gli-kruppel family member 3	*GLI3*	* 165240	7p14.1
Pre-mRNA-processing factor 4B	*PRPF4B*	* 602338	6p25.2
Protein-tyrosine phosphatase, receptor-type, delta	*PTPRD*	* 601598	9p24.1-p23
Heat-responsive protein 12	*RIDA*	* 602487	8q22.2
Transcription factor Sp4	*SP4*	* 600540	7p15.3
Tubulin, beta-3	*TUBB3*	* 602661	16q24.3
**Reference:** Rotter J, Li X, Owen LA, Taylor K, Ostmo S, Chen YI, Coyner A, Sonmez K, Hartnett ME, Guo X, Ipp E, Roll K, Genter P, Chan RVP, DeAngelis M, Chiang M, Campbell JP. Genome-wide association identifies novel ROP risk loci in a multi-ethnic cohort. Res Sq [Preprint]. **2023**:rs.3.rs-2855404. doi: 10.21203/rs.3.rs-2855404/v1. Update in: Commun Biol. 2024;7(1):107. doi: 10.1038/s42003-023-05743-9. PMID: 37292936; PMCID: PMC10246102. Li X., Owen L.A., Taylor K.D. *et al.* Genome-wide association identifies novel ROP risk loci in a multiethnic cohort. *Commun Biol* 7, 107 (2024). https://doi.org/10.1038/s42003-023-05743-9			
